# Clinical Reports

**Published:** 1893-03

**Authors:** W. T. Bull

**Affiliations:** New York; Anfputation of Arm


### CLINICAL REPORTS.

### NEW YORK HOSPITAL. SERVICE OF DR. W. T. BULL. AMPUTATION OF ARM.

The patient was a laboring man, forty-eight years old. Two months ago he received a severe laceration of the fore-arm and hand, for which he underwent treatment at a dispensary. The wound was soon neglected and an extensive cellulitis ensued, involving the hand and lower part of the arm. When he was admitted to the hospital a couple of weeks ago his pulse was 120 and his temperature 103 F. Besides the cellulitis the flexor tendons of the fingers were in a sloughing condition, and there was a suppurative arthritis of the wrist joint. The condition of the hand at this time indicated the necessity of an amputation, and it was feared that the shock of an operation while his constitutional symptoms were so pronounced would result fatally. Consequently a conservative plan was adopted. The arm was elevated in a sling and the injured part subjected

* Reported expressly for SOUTHERN MEDICAL RECORD by E. Dolan.

to continuous irrigation with bi-chloride solution. The patient's condition was thus much improved. His appetite returned, he gained in flesh, and his pulse and temperature went down to nearly normal. The suppuration of the hand, while not controlled, was lessened, the inflammation subsided and the cellulitis of the fore-arm nearly disappeared. The sloughing of the tendons still continued and the arthritis had so effected the wrist joint that discharging sinuses opened from it. The circulation of the hand was so seriously impaired that it was evident gangrene would soon result. Such was the condition of things at the time of the operation. By having waited until his constitutional symptoms had abated the danger from shock was lessened as well as having reduced the cellulitis so that the arm could be amputated at a lower point than it otherwise could.

The patient was anæsthetized, and, owing to the continued septic processes to which the parts had been subject, the wound was irrigated with a 1 to 1,000 bichloride solution. On opening into the wrist joint the lower ends of the radius and ulna were found to be undergoing a process of necrosis, consequently the arm was amputated one inch above the wrist joint. It was noticed that upon the removal of the esmarc the tissues did not retract as is usually the case. This was due to the long continued effects of the cellulitis, which, to a slight extent, still existed. A healthy flap will retract about one-third its length. As the parts were not in a healthy condition, primary intention was not expected. The wound was irrigated, sutures inserted, and the stump subjected to pressures to stop the oozing from the small arteries, the large ones having been ligated before the removal of the esmarc.

CASTRATION FOR TUBERCULAR TESTIS.

The patient was thirty-five years old, and for some time, has been under treatment for suppuration in the right side of the scrotum. He never had any venereal or constitutional disease. Three months ago his right testicle began to swell and give slight pain. There were no symptoms of any infection or inflammation extending to the bladder or ureters. The swelling became soft two weeks ago. At this time an incision was made into it and pus discharged. An open granulating wound formed and since that time pus has constantly been discharged. Thê body of the right testicle was considerably enlarged, as was also the epididymis of the diseased testicle. The left testicle was not involved. The cord on the right side was normal as were also the inguinal glands, the prostate and urethra.

The great majority of suppurating lesions in the testicle or epdidymis are tubercular ; it is rare for these organs to suppurate from traumatism or venereal diseases, although a gumma of the body of the testicle may cause suppuration. This case was diagnosed as tubercular, which diagnosis was confirmed by finding tubercle vacilli in the pus that was discharged from the sinus.

A suppurating lesion in this region runs about the same course as it does elsewhere, except that its course is more rapid. While it it is apt to break down and be absorbed, it may undergo fibroid changes. It soón involves the vas deferens of the same side then extends to the opposite testicle and its vas deferens. From here it extends to the prostate, seminal vesicle, bladder and kidneys, thus soon causing death. The cause may not be as unfavorable as this, but it usually runs a rapid course and ends fatally. The proper treatment is to arrest this threatened invasion of surrounding organs by an early removal of the primary lesion. In this case such could only be done by removal of the testicle.

The hair of the pubes, penis and perineun was removed and the parts thoroughly washed. The skin of the scrotum was made tense and an incision made from a point just below the external abdominal ring to the lower end of the scrotum. This was deepened down to the tunica vaginalis. Inflammatory adhesions that had formed were dissected away and the testicle with its tunic rolled out of the scrotum. The cord was then cut and allowed to retract after its three arteries had been ligated. There is little danger of hemorrhage here, old editions of surgery to the contrary notwithstanding. The part of the vas deferens that remained was found not to be involved. The wound was closed with interrupted catgut sutures, and as it was clean, it was not thought necessary to employ drainage. The parts were dressed with iodoform.

Upon examination the removed testicle was found to contain a suppurating focus with several other infiltrated points of tuberculous granulations nearly ready to suppurate.

The wound will heal rapidly and the patient can be discharged in a week, until which time the dressing will not be disturbed unless evidence of wound reaction, as fever and pains ensue, when the wound will be opened and drained immediately.

Usually a man doesn't like to submit to castration, thinking that such a procedure may influence future paternity. Such a patient should be instructed that by the removal of one testicle the other may be preserved. A tuberculous testicle should not be expected to recover spontaneously, and by its early removal one testicle may be preserved. When the disease is far advanced and includes the spermatic cords and prostate it may be proper to castrate, but a cure is rarely thus effected although the advance of the disease may be checked. Involvment of the lungs and phthisis may thus be prevented.

AMPUTATION OF CHEEK FOR EPITHELIOMA.

Patient was a man, laborer, forty-five years old. Five years ago he noticed a little warty growth on the inner side of his right cheek. This growth was much irritated by his chewing tobacco. This habit might have been the cause of the growth. A number of similar growths soon formed, which gradually fused into one, and formed a large oval patch. As it increased in size it was attended with ulceration which rendered his saliva turbid and gave a foul odor to his breath. His general health has been good.

Examination of his face and neck gave a suspicion of glandular enlargement. This had an important bearing, for if these glands were involved the operation of removing the epithelioma would probably not save him from death by cancer. The patient was informed that the operation must necessarily deform his cheek and mouth, and of the possibility of injury to the facial nerve, thus causing a partial loss of function of the orbicularis oris and dribbling of saliva.

A curved incision was made extending from the angle of the mouth to a point on the cheek about two inches to the right of the mouth, its concavity looking upward. Another incision was made, beginning and ending at the same points with its concavity looking downward. These two incisions included a circular area of tissue about two inches in diameter. This was removed including the entire thickness of the cheek. The vessels were tied before the mucous membrane was removed, thus preventing blood from entering the larynx. Both ends of the facial artery were ligated. This is the most significant artery here, although the others were also ligated. When the excised parts were removed, the patient's head was turned to the side being operated upon, in order to facilitate the flow of blood into the external world. The growth was inspected when removed, and found to have quite a large healthy area on all sides of it, excepting the upper; consequently a second incision was made, and a small area of tissue removed from the upper part of the wound. A surgeon, who spans tissue in a case of this kind, is placing his patient's life in jeopardy.

Deep sutures were now inserted down near but not through the mucous membrane, which, because of its distensibility, could be brought together. This was to keep the secretions of the mouth from interfering with the wound and preventing healing. A rubber drainage tube was inserted and the skin brought together and sutured. In order to get the edges of skin together it was found necessary to dissect the tissues away from the lower jaw, thus making the soft parts more distensible. If the tissues around the sutures become white, thus indicating that the tension is too great, the sutures will be removed or an incision made in the skin a little below the line of sutures.

An incision was then made extending down the neck over the carotid artery. The lymphatic glands in this region were found to be involved and were harder and whiter than normal. After their removal this incision was closed. A dressing of iodoform gauze, saturated with tincture of benzoate, and gummed, was put in the mouth on the inner side of the wound, and the external wound dressed with moist gauze and bandaged.

Had this disease progressed a few months longer the lower jaw would have deen involved. As it is there would have been less tension had a part of it been removed. A possible disadvantage of the operation is the embarassment that will result in attempting to open the mouth ; this condition can be relieved by a subsequent operation. As one angle of his mouth was taken away, and a large part of the cheek, his face will necessarily be considerably distorted. This can be partially hidden by his growing a beard.

This patient is not entitled to a good prognosis because of the involved glands. This shows that the cancerous elements are emmigrating. The fact that had that growth been removed four or five years ago his death from cancer would have been avoided, impresses the necessity of attending to these growths early. The surgical interest and instruction connected with this case has a melancholy aspect thrown over it by the sad prospect of the patient.

EXCISION OF COCCYX.

Patient was a female, twenty-three years old, by occupation a dressmaker. Nine years ago she fell on the lower end of the spine. Since that time she had been troubled with neuralgic pains from time to time. About a year ago the pain became severe, and of late, has incapacitated her for work. It was more pronounced when rising after being in the sitting posture for an hour or two or when she was in the stooping position. There has been no pain on defecation and her constitutional condition is good. On examination it revealed that there was a blunt ending to the spine as if the coccyx were placed crosswise on the sacrum, or were broken in its center and the lower fragment pointing backward. Clinical experience shows that fracture of the coccyx is followed by obstinate neuralgic pains which are only relieved by removal of the bone.

The patient was anæsthetized, placed on her abdomen and the parts shaved and washed. A longitudinal incision was made about two inches long over the coccyx extending down to the bone. The soft tissues around the coccyx were dissected from it, taking care not to injure the rectum. The bone was then seized with a bone-forceps and pulled out. The wound was about two inches deep. A bleeding vessel in the bottom of the wound, being too deep seated to easily tie, was controlled by torsion. A couple of deep sutures were put in and the wound packed with iodoform gauze and left to heal by granulation, the sutures not approximating the surfaces, but holding the packing in position. A narrower and shallower pad will replace the first one that was put in, in three days, thus giving the wound a chance to heal.

Upon examination the coccyx was found to have been fractured at the junction of its middle with its upper third and the lower fragment directed backward, firmly attached to the upper fragment at a right angle. While the operation is compara tively simple, it is found difficult to obtain rapid healing. The operator has done this operation about half a dozen times and has never had union by first intention. As a rule the patient must remain in bed two weeks, and after that time, have the wound dressed every day for about a month, the same as the after-treatment for an operation for fistula in ano. The failure to secure rapid union is due to the difficulty in keeping the wound clean as well as to the fact that it is in a locality where it is hard to keep a dressing properly applied.

